# A Synchronous-Asynchronous Particle Swarm Optimisation Algorithm

**DOI:** 10.1155/2014/123019

**Published:** 2014-07-10

**Authors:** Nor Azlina Ab Aziz, Marizan Mubin, Mohd Saberi Mohamad, Kamarulzaman Ab Aziz

**Affiliations:** ^1^Faculty of Engineering, University of Malaya, 50603 Kuala Lumpur, Malaysia; ^2^Multimedia University, Jalan Ayer Keroh Lama, 75450 Bukit Beruang, Melaka, Malaysia; ^3^Faculty of Computing, Universiti Teknologi Malaysia, 81310 Johor Bahru, Malaysia

## Abstract

In the original particle swarm optimisation (PSO) algorithm, the particles' velocities and positions are updated after the whole swarm performance is evaluated. This algorithm is also known as synchronous PSO (S-PSO). The strength of this update method is in the exploitation of the information. Asynchronous update PSO (A-PSO) has been proposed as an alternative to S-PSO. A particle in A-PSO updates its velocity and position as soon as its own performance has been evaluated. Hence, particles are updated using partial information, leading to stronger exploration. In this paper, we attempt to improve PSO by merging both update methods to utilise the strengths of both methods. The proposed synchronous-asynchronous PSO (SA-PSO) algorithm divides the particles into smaller groups. The best member of a group and the swarm's best are chosen to lead the search. Members within a group are updated synchronously, while the groups themselves are asynchronously updated. Five well-known unimodal functions, four multimodal functions, and a real world optimisation problem are used to study the performance of SA-PSO, which is compared with the performances of S-PSO and A-PSO. The results are statistically analysed and show that the proposed SA-PSO has performed consistently well.

## 1. Introduction

Particle swarm optimisation (PSO) was introduced by Kennedy and Eberhart in 1995 [1]. It is a swarm-based stochastic optimisation algorithm that mimics the social behaviour of organisms such as birds and fishes. These organisms' success in looking for food source is achieved through individual effort as well as corporation with surrounding neighbours. In PSO, the individuals are represented by a swarm of agents called particles. The particles move within the search area to find the optimal solution by updating their velocity and position. These values are influenced by the experience of the particles and their social interactions. The PSO algorithm has been successfully applied in various fields, such as human tremor analysis for biomedical engineering [2, 3], electric power and voltage management [4], machine scheduling [5], robotics [6], and VLSI circuit design [7].

Since its introduction, PSO has undergone numerous evolutionary processes. Many variations of PSO have been proposed to improve the effectiveness of the algorithm. Some of the improvement involves introduction of a new parameter to the algorithm such as inertia weight [8] and constriction factor [9], while others focus on solving specific type of problems such as multiobjective optimization [10, 11], discrete optimization problems [12, 13], and dynamic optimization problems [14].

Here we focus on the effect of the particles' update sequence on the performance of PSO. In the original PSO, a particle's information on its neighbourhood's best found solution is updated after the performance of the whole swarm is evaluated. This version of PSO algorithm is known as synchronous PSO (S-PSO). The synchronous update in S-PSO provides the perfect information concerning the particles, thus allowing the swarm to choose a better neighbour and exploit the information provided by this neighbour. However, this strategy could cause the particles to converge too fast.

Another variation of PSO, known as asynchronous PSO (A-PSO), has been discussed by Carlisle and Dozier [15]. In A-PSO, the best solutions are updated as soon as a particle's performance has been evaluated. Therefore, a particle's search is guided by the partial or imperfect information from its neighbourhood. This strategy leads to diversity in the swarm [16], wherein the particles updated at the beginning of an iteration use more information from the previous iteration while particles at the end of the iteration are updated based on the information from the current iteration [17]. In several studies [15, 16, 18], A-PSO has been claimed to perform better than S-PSO. Xue et al. [19] reported that asynchronous updates contribute to a shorter execution time. Imperfect information due to asynchronous updates causes the information of the current best found solution to be communicated to the particles more slowly, thus encouraging more exploration. However, a study conducted by Juan et al. [20] reported that S-PSO is better than A-PSO in terms of the quality of the solution and also the convergence speed. This is due to the stronger exploitation.

The synchronicity of the particles influences exploration and exploitation among the particles [17]. Exploration and exploitation play important roles in determining the quality of a solution. Exploration in asynchronous update ensures that the search space is thoroughly searched so that the area containing the best solution is discovered. However, exploitation in synchronous update helps to fine tune the search so that the best solution can be found. Hence, in this paper, we attempt to improve the PSO algorithm by merging both synchronous and asynchronous updates in the search process so that the advantages of both methods can be utilised. The proposed algorithm, which is named as the synchronous-asynchronous PSO (SA-PSO), divides the particles into smaller groups. These groups are updated asynchronously, while members within the same group are updated synchronously. After the performance of all the particles in a group is evaluated, the velocities and positions of the particles are updated using a combination of information from the current iteration of their own group and the groups updated before them, as well as the information from the previous iteration of the groups that have not yet been updated. The search for the optimal solution in SA-PSO is led by the groups' best members together with the swarm's best. This strategy is different from the original S-PSO and A-PSO, where the search is led by the particles' own experience together with the swarm's best.

The rest of the paper is organised as follows. The S-PSO and A-PSO algorithms are discussed in Section 2. The proposed SA-PSO algorithm is described in detail in Section 3. In Section 4, the performance of the SA-PSO algorithm is evaluated using ten benchmark functions comprising of five unimodal functions, four multimodal functions, and a real world optimisation problem. The results of the tests are presented and discussed in Section 5. Our conclusions are presented in Section 6.

## 2. Particle Swarm Optimisation

### 2.1. Synchronous PSO

In PSO, the search for the optimal solution is conducted by a swarm of *P* particles. At time *t*, the *i*th particle has a position, *x*_*i*_(*t*), and a velocity, *v*_*i*_(*t*). The position represents a solution suggested by the particle while velocity is the rate of change from the current position to the next position. At the beginning of the algorithm, these two values (position and velocity) are randomly initialised. In subsequent iterations, the search process is conducted by updating the position and velocity using the following equations:

(1) vi(t)=ωvi(t−1)+c1r1(pBesti−xi(t−1))+c2r2(gBest−xi(t−1)),

(2) xi(t)=vi(t)+xi(t−1).

To prevent the particles from venturing too far from the feasible region, the *v*_*i*_(*t*) value is clamped to ±*V*_max⁡_. If the value of *V*_max⁡_ is too large, then the exploration range is too wide. Conversely, if the value of *V*_max⁡_ is too small, then the particles will favour the local search [21]. In (1), *c*_1_ and *c*_2_ are the learning factors that control the effect of the cognitive and social influence on a particle. Typically, both *c*_1_ and *c*_2_ are set to 2 [22]. Two independent random numbers *r*_1_ and *r*_2_ in the range [0.0, 1.0] are incorporated into the velocity equation. These random terms provide stochastic behaviour to the particles, thus encouraging them to explore a wider area. Inertia weight, *ω*, which is a term added to improve the PSO's performance, controls the particles' momentum. When a good area is found, the particles can switch to fine tuning by manipulating *ω* [8]. To ensure convergence, a time decreasing inertia weight is more favourable than a fixed inertia weight [21]. This is because a large inertia weight at the beginning helps to find a good area through exploration and a small inertia weight towards the end—when typically a good area is already found—facilitates fine tuning. The small inertia weight at the end of the search reduces the global search activity [23].

An individual success in PSO is affected not only by the particle's own effort and experience but also by the information shared by its surrounding neighbours. The particle's experience is represented in (1) by *p*Best_*i*_, which is the best position found so far by the *i*th particle. The neighbours' influence is represented by *g*Best, which is the best position found by the swarm up to the current iteration.

The particle's position, *x*_*i*_(*t*), is updated using (2), in which a particle's next search is launched from its previous position and the new search is influenced by the past search [24]. Typically, *x*_*i*_(*t*) is bounded to prevent the particles from searching in an infeasible region [25]. The quality of *x*_*i*_(*t*) is evaluated by a problem-dependent fitness function. Each of the particles is evaluated to determine its current fitness. If a new position with a better fitness than the current fitness of *g*Best or *p*Best_*i*_ or both is found, then the new position value will accordingly be saved as *g*Best or *p*Best_*i*_; otherwise the old best values will be adopted. This update process continues until the stopping criterion is met, when either the maximum iteration limit, *T*, is achieved or the target solution is attained. Therefore, for a swarm with *P* number of particles, the maximum number of fitness evaluation in a run is (*P* × *T*).

**Figure 1:**
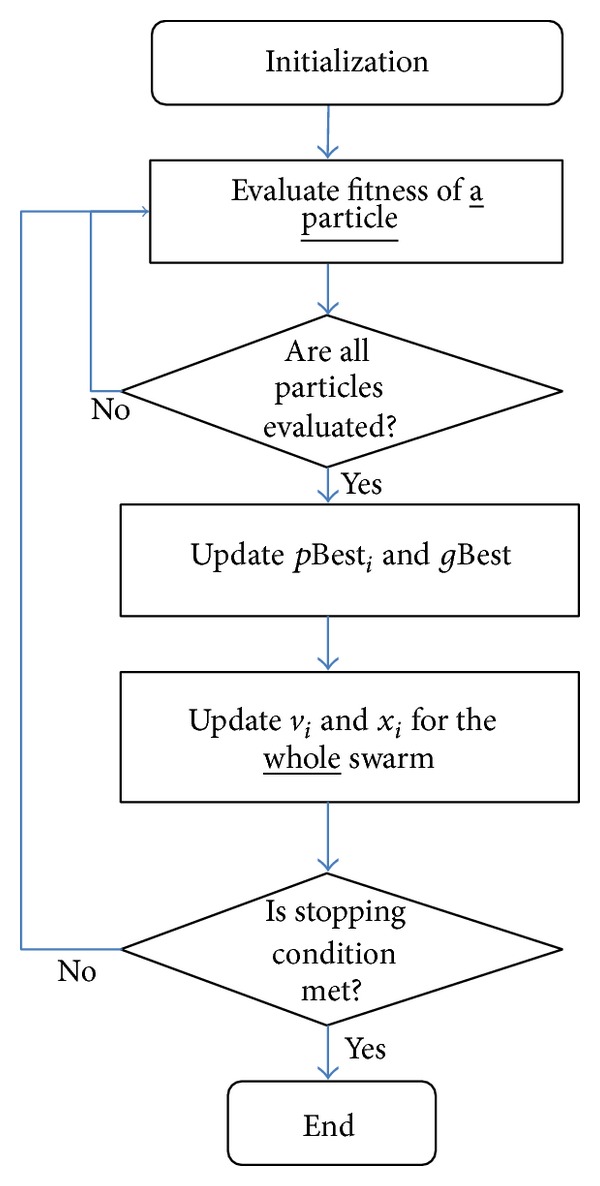
S-PSO flowchart.

The original PSO algorithm is shown in the flowchart of Figure 1. As shown in the algorithm, the particles' *p*Best_*i*_ and *g*Best updates are conducted after the fitness of all the particles has been evaluated. Therefore, this version of PSO is known as synchronous PSO (S-PSO). Because the *p*Best_*i*_ and *g*Best are updated after all the particles are evaluated, S-PSO ensures that all the particles receive perfect and complete information about their neighbourhood, leading to a better choice of *g*Best and thus allowing the particles to exploit this information so that a better solution can be found. However, this possibly leads the particles in S-PSO to converge faster, resulting in a premature convergence.

### 2.2. Asynchronous PSO

In S-PSO, a particle must wait for the whole swarm to be evaluated before it can move to a new position and continue its search. Thus, the first evaluated particle is idle for the longest time, waiting for the whole swarm to be updated. An alternative to S-PSO is A-PSO, in which the particles are updated based on the current state of the swarm. A particle in A-PSO is updated as soon as its fitness is evaluated. The particle selects *g*Best using a combination of information from the current and the previous iteration. This is different from S-PSO, in which all the particles use information from the same iteration. Consequently, in A-PSO, particles of the same iteration might use various values of *g*Best, as it is selected based on the available information during a particle's update process.

**Figure 2:**
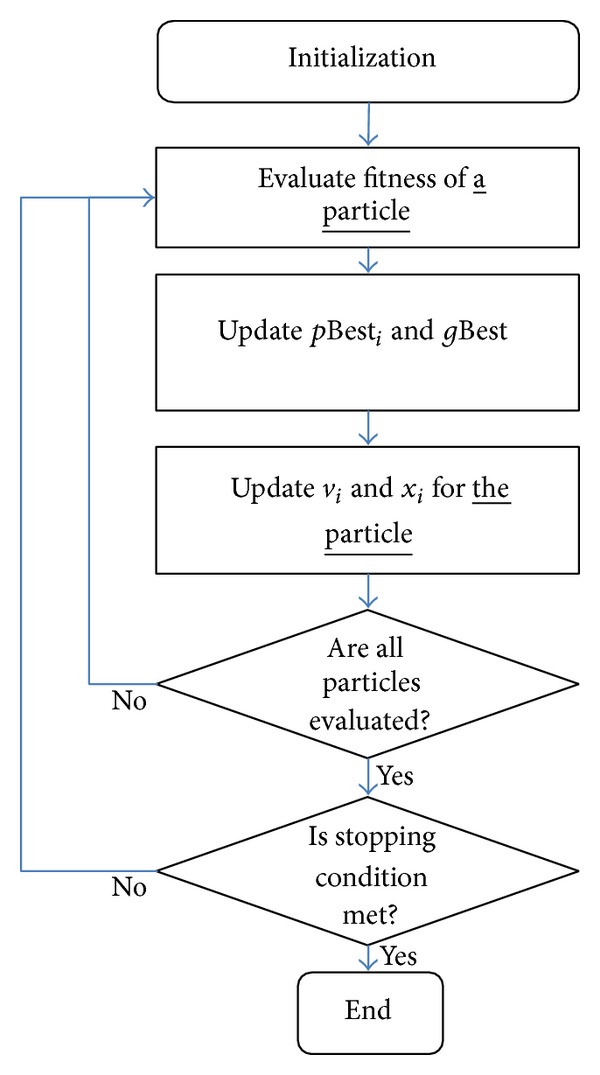
A-PSO flowchart.

The flowchart in Figure 2 shows the A-PSO algorithm. The flow of A-PSO is different than S-PSO; however the fitness function is still called for *P* times per iteration, once for each particle. Therefore, the maximum number of fitness evaluation is (*P* × *T*). This is similar to S-PSO. The velocity and position are calculated using the same equations as S-PSO.

Other than the variety of information, the lack of synchronicity in A-PSO solves the issue of idle particles faced in S-PSO [26]. An asynchronous update also enables the update sequence of the particles to change dynamically or a particle to be updated more than once [26, 27]. The change in the update sequence offers different levels of available information among the particles, and such differences can prevent the particles from being trapped in local optima [17].

## 3. The Proposed Synchronous-Asynchronous PSO (SA-PSO)

In this paper, the PSO algorithm is improved by merging both update methods. The proposed algorithm, synchronous-asynchronous PSO (SA-PSO), divides the particles into smaller groups. In S-PSO and A-PSO, the particles learn from their own best experience, *p*Best_*i*_^*c*^ and *g*Best. However, in the proposed algorithm, instead of using their own experience, the particles learn from their group's performance.

**Figure 3:**
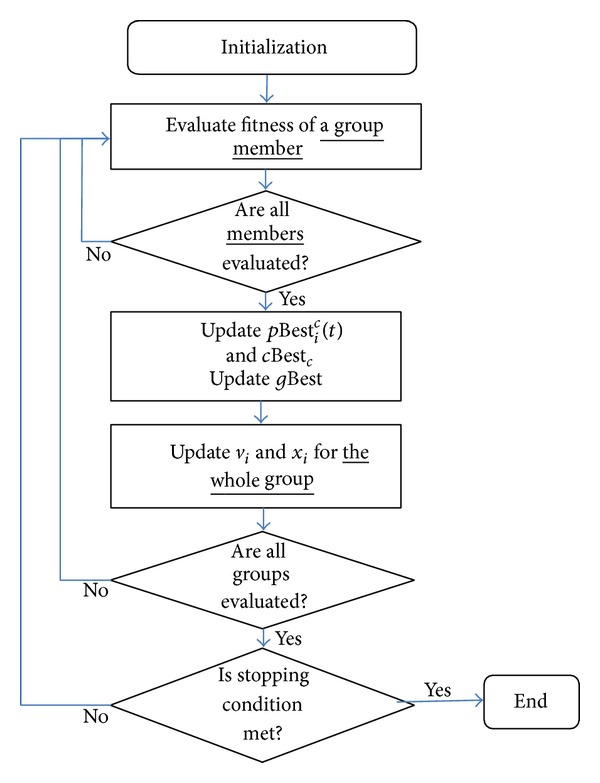
SA-PSO flowchart.

The algorithm proposed is presented in the flowchart shown in Figure 3. The algorithm starts with initialisation of particles. The particles in SA-PSO are divided into *C* groups, each of which consists of *N* number of particles. Initially, *C* central particles, one for each group, are randomly initialized within the search space. This is followed by random placement of *N* − 1 number of members for each group. The distances of members are within the radius of ±Δ from the central particle of their respective groups. Therefore, Δ is the maximum distance of a particle from the central particle of its group. This parameter is only used once throughout the execution of the algorithm—during the initialisation phase. Group memberships remain fixed throughout the search process. The total number of particles, *P*, is *C* × *N* for the SA-PSO algorithm.

The groups are updated one by one; that is, asynchronous update is used across groups. The particles from the group that is being updated use three groups of information to update their velocity. The first group of information is the current information of the particles' group members; the particles use this information to try to match their group's best performer. The particles also use recent information from the groups that were updated earlier and information from the previous iteration for the groups to be updated later.

When a group is updated, the group members' velocity and position updates are performed after the whole group performance is evaluated. Therefore, the particles in a group are updated synchronously.

When a group evaluates the performance of its members, the fitness function is called for *N* times. One by one of the groups' members are updated in an iteration. Since there is *C* number of groups, hence the fitness function is called for *C* × *N* times, which is equivalent to *P* times per iteration. Therefore, although the particles in SA-PSO are divided into groups, the maximum number of fitness evaluation per run is the same as S-PSO and A-PSO which is (*P* × *T*).

The velocity at time *t* of *i*th particle that belongs to *c*th group, *v*_*i*_^*c*^(*t*), is updated using the following equation:

(3) vic(t)=ωvic(t−1)+c1r1(cBestc−xic(t−1))+c2r2(gBest−xic(t−1)).

Equation (3) shows that the information used to update the velocity are *c*Best_*c*_ and *g*Best. *c*Best_*c*_ is the best member of *c*th group, where *c* is [1, *C*], and it is chosen among the particle's best of *c*th group, *p*Best_*i*_^*c*^. This value, together with the swarm's best, *g*Best, leads the particles' search in the SA-PSO algorithm. The *g*Best is updated after all once a new *c*Best_*c*_ outperforms *g*Best. Thus, *g*Best is the best *c*Best_*c*_. The communication among the groups in SA-PSO is conducted through the best performing member of the groups. The position of the particle, *x*_*i*_^*c*^(*t*), is updated using

(4) $$\begin{array}{c} x_{i}^{c}\left(t\right)=v_{i}^{c}\left(t\right)+x_{i}^{c}\left(t-1\right). \end{array}$$

The algorithm is ended when either the ideal fitness is achieved or maximum iteration is reached.

The SA-PSO algorithm takes advantage of both A-PSO and S-PSO algorithms. In A-PSO, the particles are updated using imperfect information, which contributes to the diversity and exploration. In S-PSO, the quality of the solution found is ensured by evaluating the performance of the whole swarm first. The S-PSO particles are then updated by exploiting this information. The asynchronous update characteristic of A-PSO is imitated by SA-PSO by updating the groups one after another. Hence, members of a group are updated using the information from mixed iterations. This strategy encourages exploration due to the imperfect information. However, the performance of all members of a group in SA-PSO is evaluated first before the velocity and position update process starts. This is the synchronous aspect of SA-PSO. It provides the complete information of the group and allows the members to exploit the available information.

## 4. Experiments

The proposed SA-PSO and the existing S-PSO and A-PSO were implemented using MATLAB. The parameter settings are summarised in Table 1. Each experiment was subjected to 500 runs. The initial velocity was set to random value subject to the velocity clamping range, ±*V*_max⁡_. The position of the particles was randomly initialised within the search space. A linear decreasing inertia weight ranging from 0.9 to 0.4 was employed to encourage fine tuning towards the end of the search. The cognitive and social learning factors were set to 2 which is a typical value for *c*_1_ and *c*_2_. The search was terminated either due to the number of iterations reaching 2000 or the ideal solution being found. The maximum number of iteration is set to 2000 to limit the computational time taken. The final *g*Best values were recorded. The setting for the additional parameters in SA-PSO is given in Table 2. Exclusively for SA-PSO, the members of the groups were randomly initialised with their distance to group centres, Δ. The group centres were randomly initialised within the boundary of the search space.

A group of benchmark test problems had been identified for assessing the performance of the proposed SA-PSO and the original S-PSO and A-PSO algorithms. The benchmark test problems consist of five unimodal functions, four multimodal functions, and one real world optimisation problem, namely, frequency-modulated (FM) sound wave synthesis which is taken from CEC2011 competition on testing evolutionary algorithms on real world optimisation problems [28]. These functions are given in Table 3. All functions used are minimisation functions with ideal fitness value of *f*(*x*) = 0. The dimension of the unimodal and multimodal problems, *n*, was set to 30. The search spaces for these problems are therefore high dimensional [29, 30]. Note that the FM sound wave problem is a six-dimensional problem.

The solutions found by the algorithms tested are presented here using boxplot. A boxplot shows the quality and also the consistency of an algorithm's performance. The size of the box shows the magnitude of the variance of the results; thus a smaller box suggests a consistent performance of the algorithm. Because the benchmark functions used in this study are minimisation problems, a lower boxplot is desirable as it indicates better quality of the solutions found.

The algorithms are compared using a nonparametric test due to the nature of the solutions found, where they are not normally distributed. The test chosen is the Friedman test with significance level *α* = 0.05. This test is suitable for comparison of more than two algorithms [31]. The algorithms are first ranked based on their average performance for each benchmark function. The average rank is then used to calculate the Friedman statistic value. According to the test, if the statistic value is lesser than the critical value, the algorithms tested are identical to each other; otherwise, significant differences exist. If a significant difference is found, the algorithms are then compared using a post hoc procedure. The chosen post hoc procedure here is the Holm procedure. It is able to pinpoint the algorithms that are not identical to each other, a result that cannot be detected by the Friedman test.

## 5. Results and Discussion

### 5.1. SA-PSO versus S-PSO and A-PSO

**Figure 4:**
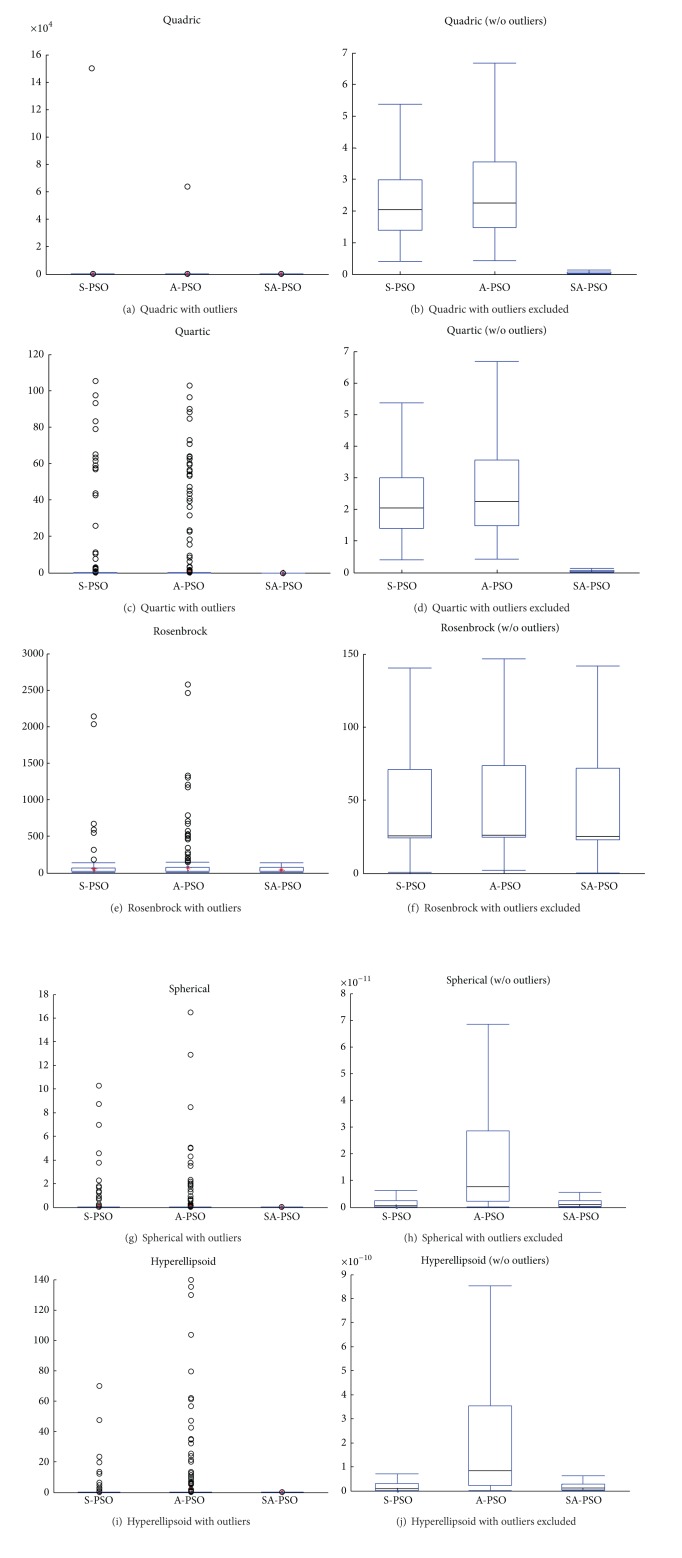
Results of experiments on unimodal functions.

The boxplots in Figure 4 show the quality of the results for unimodal test functions using the three algorithms. The results obtained by S-PSO and A-PSO algorithms contain multiple outliers. These out-of-norm observations are caused by the stochastic behaviour of the algorithms. The proposed SA-PSO exhibits no outliers for the unimodal test functions. The particles in SA-PSO are led by two particles with good experience, *g*Best and *c*Best_*c*_, instead of *g*Best only like S-PSO and A-PSO. Learning from *c*Best_*c*_ of each group reduces the effect of the stochastic behaviour in SA-PSO.

The presence of the outliers makes it difficult to observe the variance of the results through the box plot. Therefore, the outliers are trimmed in the boxplots of Figures 4(b), 4(d), 4(f), 4(h), and 4(j). The benchmark functions tested here are minimisation functions; hence, a lower boxplot indicates better quality of the algorithm. It can be observed from the figure that SA-PSO continuously gives good performance in all the unimodal functions tested. The sizes of the boxplots show that the SA-PSO algorithm provides a more consistent performance with smaller variance.

**Figure 5:**
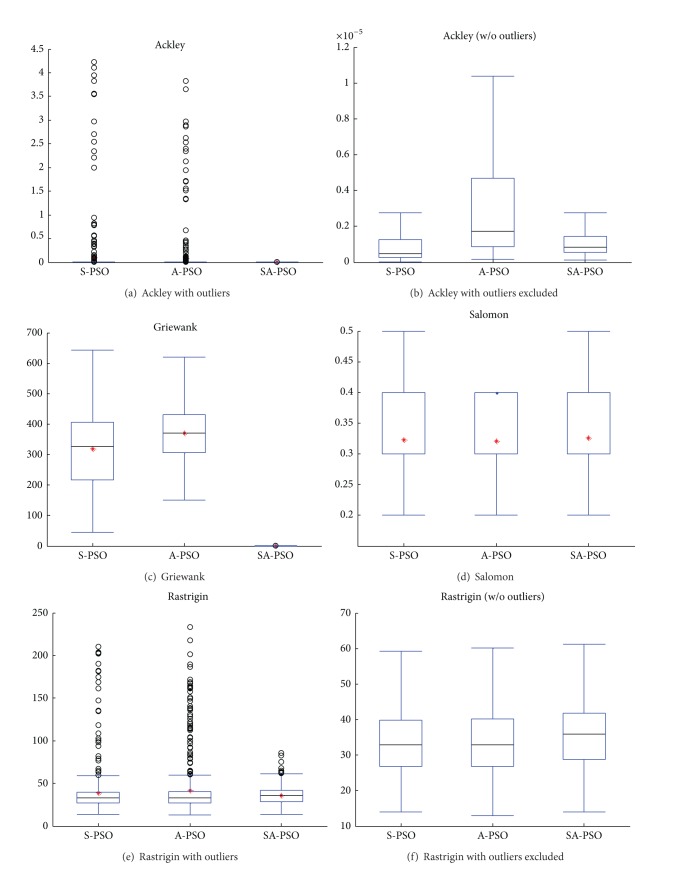
Results of experiments on multimodal functions.

The results of the test on multimodal problems are shown in the boxplots in Figure 5. S-PSO and A-PSO have outliers for Ackley and Rastrigin while SA-PSO only has outliers in the results of Rastrigin. The Rastrigin function has a nonprotruding minima, which complicates the convergence [32]. However, SA-PSO has fewer outliers compared to S-PSO and A-PSO. This observation once again proves the efficiency of learning from two good particles, *g*Best and *c*Best_*c*_.

Similar to the boxplots for unimodal test functions, the boxplots, after trimming of the outliers, show that the variance of the solutions found by SA-PSO is small. The variance proves the consistency of SA-PSO's performance. SA-PSO found much better results for the Griewank function compared to the other two algorithms.

**Figure 6:**
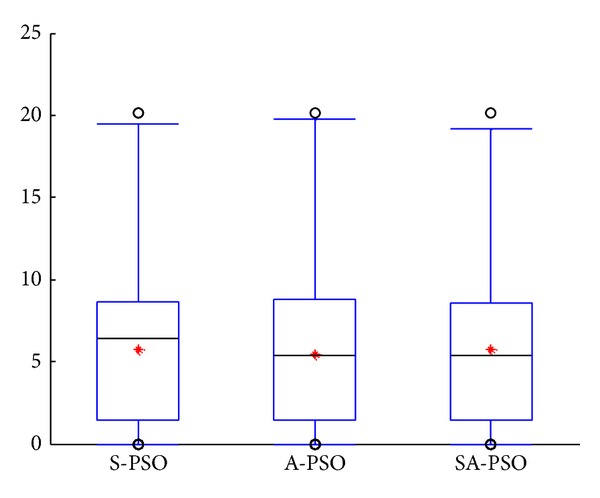
Results of experiments on parameter estimation for FM sound wave.

The three algorithms tested have similar performance for the FM sound wave parameter estimation problem as shown in Figure 6. However, from the distribution of the solution in the boxplot, it could be seen that SA-PSO and A-PSO have slightly better performance than S-PSO as more solutions found are at the lower part of the box.

In Table 4, the Friedman test is conducted to analyse whether significant differences exist between the algorithms. The performances of the algorithms for all test functions are ranked based on their mean value. The means used here are calculated inclusive of the outliers because the outliers are genuine outliers that are neither measurement nor clerical errors and are therefore valid solutions. The means are shown in the boxplots (before trimming of outliers) using the ∗ symbol. According to the Friedman test, SA-PSO ranked the best among the three algorithms. The Friedman statistic value shows that significant differences exist between the algorithms. Therefore, the Holm procedure is conducted, and the three algorithms are compared against each other. The results in Table 5 show that there is significant difference between SA-PSO and the A-PSO algorithm. The Holm procedure also shows that the performance of SA-PSO is on a par with S-PSO.

### 5.2. Effect of SA-PSO Parameters

The number of particles can influence the size and the number of groups. To study the effect of these parameters, the number of particles is varied from 20 to 50. Only test functions one to nine are used here as they have similar dimension. There are 7 experiments conducted each for size of the groups and number of groups as listed in Tables 6 and 7. In the experiments for the size of the group, the number of groups is fixed at 5 and the size of the groups is increased from 4 to 10 members. The effect of the number of groups is studied using groups of 5 members; the number of groups is increased from 4, 5, 6, 7, 8, 9, and 10.

**Figure 7:**
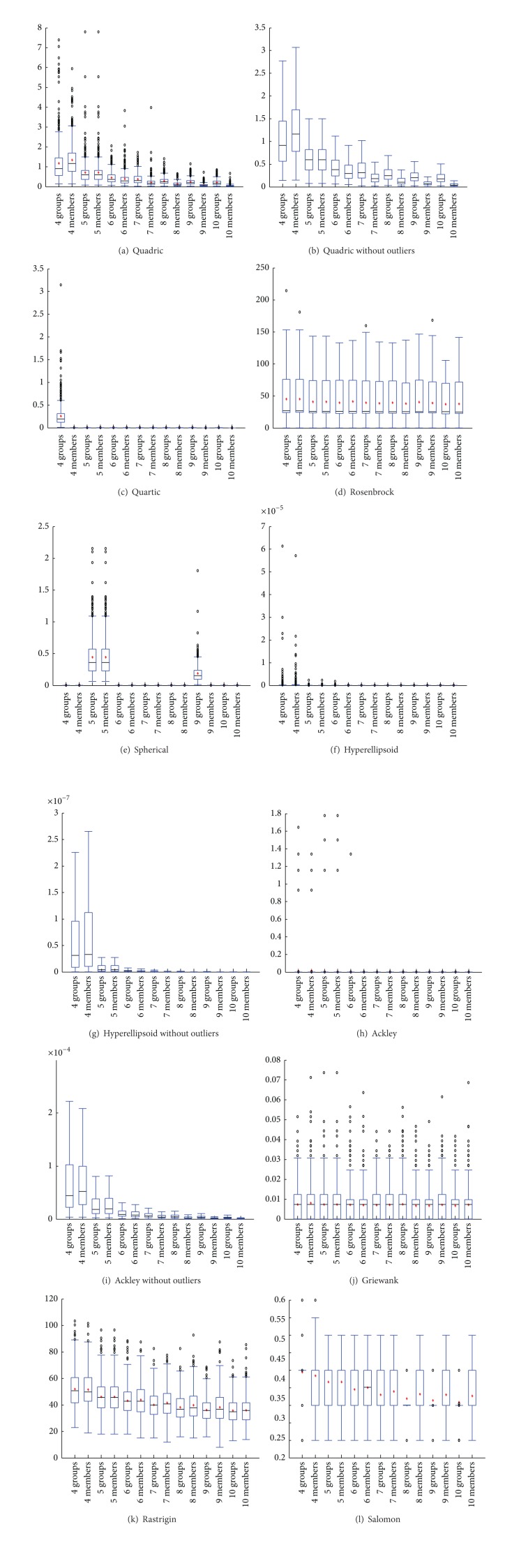
Effect of number of groups and group size.

The average results for the effect of size of groups and number of groups are presented in Tables 8 and 9. Generally the results show that, similar to the original PSO algorithm, the number of particles affects the performance of SA-PSO. A higher number of particles, that is, bigger groups or higher number of groups, contributes to a better performance. However, the effect is also influenced by the test function. This can be observed in Figure 7, for quadric and Ackley functions, the effect is more obvious compared to other functions.

Friedman test is performed on the experimental results in Tables 8 and 9. The test is conducted to study the effect of number of group and group's size on SA-PSO's performance. The average rank is presented in Table 10.

The result of Friedman test shows that significant difference exists in the SA-PSO performance for different number of groups. Hence, Holm procedure is conducted and its statistical values are tabulated in Table 11. The result of the Holm procedure shows that significant differences exist between SA-PSO implementations if the populations in each implementation consist of unequal number of groups and the difference in the number of groups is greater than three.

The Friedman test performed on the effect of the group size shows that the SA-PSO implemented with groups of different sizes are significantly different. This observation is further studied using Holm procedure as in Table 12. The outcome of Holm procedure reveals that significant difference exists between two implementations of SA-PSO algorithm if the difference in the group size is greater than three particles.

Δ is a new parameter introduced in SA-PSO. It represents the maximum distance of a particle to its group head during the initialisation stage of the algorithm. The value of Δ determines the distribution of the particles within the search space. A small Δ will result in close groups, while a large Δ will result in groups with a bigger radius. The effect of Δ is tested here, and the test parameters are listed in Table 13. For each of the test functions, the Δ value is set to 1%, 5%, 10%, 50%, and 100% of the length of the search space. The average performance for different values of Δ is listed in Table 14.

**Figure 8:**
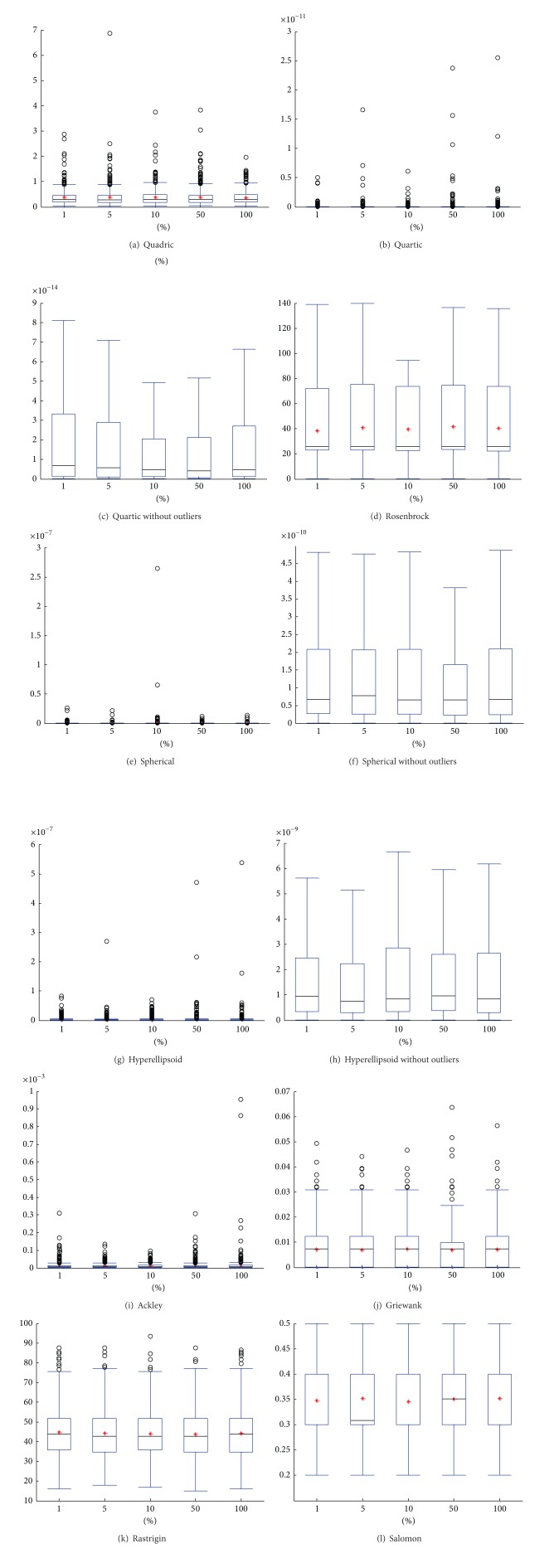
Effect of Δ.

The Friedman statistic shows that using different Δ values makes no significant difference to SA-PSO, thus showing that the performance of SA-PSO is not greatly affected by the choice of Δ. This result is confirmed by boxplots in Figure 8 where the sizes of the box in most of the test functions are similar to each other.

## 6. Conclusion

A synchronous-asynchronous PSO algorithm (SA-PSO) is proposed in this paper. The particles in this algorithm are updated in groups; the groups are updated asynchronously—one by one—while particles within a group are updated synchronously. A group's search is led by the group's best performer, *c*Best_*c*_, and the best member of the swarm, *g*Best. The algorithm benefits from good exploitation and fine tuning provided by synchronous update while also taking advantage of the exploration by the asynchronous update. Learning from *c*Best_*c*_ also contributes to the good performance of the SA-PSO algorithm. Overall, the performance of the algorithm proposed is better and more consistent than the original S-PSO and A-PSO.

## Figures and Tables

**Table 1:** Parameters setting for S-PSO, A-PSO, and SA-PSO.

Parameter	Value
Number of runs for each experiment	500
Number of iterations	2000
Velocity clamping, *V*_max⁡_	4
Range of inertia weight, *ω*	0.9–0.4
Learning factors	
*c*_1_	2
*c*_2_	2

**Table 2:** Parameters setting for the additional parameters in SA-PSO.

Parameter	Value
Number of groups, *C*	5
Group size (particles per group)	10
Initial distance to group centre, Δ	50% of the length of the search space

**Table 3:** Test functions.

Function type	Function name	Equation
Unimodal	Quadric	$f_{1}\left(x\right)=\overset{n}{\underset{i=1}{\sum }}{\left(\overset{i}{\underset{j=1}{\sum }}x_{j}\right)}^{2}$ where −100 ≤ *x*_*j*_ ≤ 100
	Quartic	$f_{2}\left(x\right)=\overset{n}{\underset{i=1}{\sum }}ix_{i}^{4}$ where −1.28 ≤ *x*_*i*_ ≤ 1.28
	Rosenbrock	$f_{3}\left(x\right)=\overset{n-1}{\underset{i=1}{\sum }}\left[100{\left(x_{i}^{2}-x_{i+1}\right)}^{2}+{\left(x_{i}-1\right)}^{2}\right]$ where −2.048 ≤ *x*_*i*_ ≤ 2.048
	Spherical/De Jong's	$f_{4}\left(x\right)=\overset{n}{\underset{i=1}{\sum }}x_{i}^{2}$ where −5.12 ≤ *x*_*i*_ ≤ 5.12
	Hyperellipsoid	$f_{5}\left(x\right)=\overset{n}{\underset{i=1}{\sum }}ix_{i}^{2}$ where −5.12 ≤ *x*_*i*_ ≤ 5.12

Multimodal	Ackley	$f_{6}\left(x\right)=20+e-20\exp\left[-0.2\sqrt{\frac{1}{n}\overset{n}{\underset{i=1}{\sum }}x_{i}^{2}}\right]-\exp\left[\frac{1}{n}\overset{n}{\underset{i=1}{\sum }}\cos\left(2\pi x_{i}\right)\right]$ where −32.768 ≤ *x*_*i*_ ≤ 32.768
	Griewank	$f_{7}\left(x\right)=1+\frac{1}{4000}\overset{n}{\underset{i=1}{\sum }}x_{i}^{2}-\prod_{i=1}^{n}\cos\left(\frac{x_{i}}{\sqrt{i}}\right)$ where −600 ≤ *x*_*i*_ ≤ 600
	Rastrigin	$f_{8}\left(x\right)=10n+\overset{n}{\underset{i=1}{\sum }}\left[x_{i}^{2}-10\cos\left(2\pi x_{i}\right)\right]$ where −5.12 ≤ *x*_*i*_ ≤ 5.12
	Salomon	$f_{9}\left(x\right)=1-\cos\left(2\pi \sqrt{\overset{n}{\underset{i=1}{\sum }}x_{i}^{2}}\right)+0.1\sqrt{\overset{n}{\underset{i=1}{\sum }}x_{i}^{2}}$ where −600 ≤ *x*_*i*_ ≤ 600

Real world problem	FM sound wave	*y*(*t*) = *x*_1_sin⁡⁡(*x*_2_ *tθ* + *x*_3_sin⁡⁡(*x*_4_ *tθ* + *x*_5_sin⁡⁡(*x*_6_ *tθ*))) *y*_0_(*t*) = (1)sin⁡⁡((5)*tθ* + (−1.5)sin⁡⁡((4.8)*tθ* + (2.0)sin⁡⁡((4.9)*tθ*))) $f_{10}\left(x\right)=\overset{100}{\underset{t=0}{\sum }}{\left(y\left(t\right)-y_{0}(t)\right)}^{2}$ where $\theta =\frac{2\pi }{100}$ and −6.4 ≤ *x*_*i*_ ≤ 6.35

**Table 4:** Friedman test on the results of the experiments.

		S-PSO	A-PSO	SA-PSO
Quadric	Mean	305.4320	131.4246	0.0537
	Friedman rank	3	2	1

Quartic	Mean	1.9524	3.0793	0.0000
	Friedman rank	2	3	1

Rosenbrock	Mean	58.7646	71.5899	37.9161
	Friedman rank	2	3	1

Spherical	Mean	0.0963	0.1628	0.0000
	Friedman rank	2	3	1

Hyperellipsoid	Mean	0.4151	2.5037	0.0000
	Friedman rank	2	3	1

Ackley	Mean	0.0941	0.0898	0.0000
	Friedman rank	3	2	1

Griewank	Mean	317.8628	371.7447	0.0071
	Friedman rank	2	3	1

Rastrigin	Mean	38.6035	42.2694	36.1207
	Friedman rank	2	3	1

Salomon	Mean	0.3227	0.3211	0.3263
	Friedman rank	2	1	3

FM sound wave	Mean	5.7751	5.4484	5.7402
	Friedman rank	3	1	2

Average Friedman rank		2.3	2.4	1.3

**Table 5:** Holm procedure on the results of the experiments.

Dataset	*z*	*P*	Holm
A-PSO versus SA-PSO	2.4597	0.0139	0.0167
S-PSO versus SA-PSO	2.2361	0.0253	0.0250
S-PSO versus A-PSO	0.2236	0.8231	0.0500

**Table 6:** Experimental setup for size of groups.

Number of particles	Size of groups
20	4
25	5
30	6
35	7
40	8
45	9
50	10

**Table 7:** Experimental setup for number of groups.

Number of particles	Number of groups
20	4
25	5
30	6
35	7
40	8
45	9
50	10

**Table 8:** Average results on the experiments involving the size of group.

Size of groups	Quadric	Quartic	Rosenbrock	Spherical	Hyperellipsoid	Ackley	Griewank	Rastrigin	Salomon
4	1.3459	5.183*E* − 11	45.3448	4.285*E* − 08	4.805*E* − 07	0.0184	0.0081	51.6422	0.3847
5	0.7224	1.289*E* − 12	41.0048	0.445	2.275*E* − 08	0.0089	0.0074	46.2869	0.3663
6	0.3932	1.925*E* − 13	41.5781	4.594*E* − 10	4.621*E* − 09	1.3387*E* − 05	0.007	43.886	0.3505
7	0.223	1.09*E* − 14	38.6543	7.226*E* − 11	7.833*E* − 10	6.9718*E* − 06	0.0072	41.6152	0.3396
8	0.1357	1.485*E* − 15	38.4704	1.637*E* − 11	2.455*E* − 10	3.5115*E* − 06	0.0068	39.9117	0.3315
9	0.0896	2.63*E* − 16	39.4469	6.376*E* − 12	8.392*E* − 11	1.9843*E* − 06	0.0073	38.2884	0.3297
10	0.0537	4.735*E* − 17	37.9161	4.086*E* − 12	2.704*E* − 11	1.1777*E* − 06	0.0071	36.1207	0.3263

**Table 9:** Average results on the experiments involving the number of groups.

Number of groups	Quadric	Quartic	Rosenbrock	Spherical	Hyperellipsoid	Ackley	Griewank	Rastrigin	Salomon
4	1.1854	0.262	45.3952	2.864*E* − 06	4.349*E* − 07	0.0167	0.0073	51.9996	0.3941
5	0.7224	1.289*E* − 12	41.0048	0.445	2.275*E* − 08	0.0089	0.0074	46.2869	0.3663
6	0.462	1.381*E* − 13	39.7459	9.038*E* − 10	9.866*E* − 09	0.0027	0.0073	43.3588	0.3453
7	0.3886	9.477*E* − 14	39.66	1.205*E* − 10	1.79*E* − 09	9.4418*E* − 06	0.0071	40.1133	0.3297
8	0.3013	2.671*E* − 14	40.0051	5.109*E* − 11	7.606*E* − 10	6.1644*E* − 06	0.0075	38.2584	0.3193
9	0.25	5.154*E* − 15	40.682	0.1889	3.668*E* − 10	4.2811*E* − 06	0.0067	36.3674	0.3141
10	0.2111	3.448*E* − 15	37.3187	1.824*E* − 11	2.134*E* − 10	3.1415*E* − 06	0.0068	35.7875	0.3093

**Table 10:** Friedman test on the effect of number of groups and group size.

Number of groups	4	5	6	7	8	9	20

Average Friedman rank	6.50	6.11	4.61	3.56	3.44	2.67	1.11

Size of groups	4	5	6	7	8	9	20

Average Friedman rank	6.89	6.00	4.78	3.89	2.67	2.56	1.22

**Table 11:** Holm procedure on the effect of number of groups.

Dataset	*P*	*z*	Holm
4 groups versus 10 groups	0.0000	5.2918	0.0024
5 groups versus 10 groups	0.0000	4.9099	0.0025
4 groups versus 9 groups	0.0002	3.7643	0.0026
6 groups versus 10 groups	0.0006	3.4369	0.0028
5 groups versus 9 groups	0.0007	3.3824	0.0029
4 groups versus 8 groups	0.0027	3.0005	0.0031
4 groups versus 7 groups	0.0038	2.8914	0.0033
5 groups versus 8 groups	0.0088	2.6186	0.0036
5 groups versus 7 groups	0.0121	2.5095	0.0038
7 groups versus 10 groups	0.0164	2.4004	0.0042
8 groups versus 10 groups	0.0219	2.2913	0.0045
6 groups versus 9 groups	0.0562	1.9094	0.0050
4 groups versus 6 groups	0.0636	1.8549	0.0056
9 groups versus 10 groups	0.1266	1.5275	0.0063
5 groups versus 6 groups	0.1408	1.4730	0.0071
6 groups versus 8 groups	0.2519	1.1456	0.0083
6 groups versus 7 groups	0.3000	1.0365	0.0100
7 groups versus 9 groups	0.3827	0.8729	0.0125
8 groups versus 9 groups	0.4450	0.7638	0.0167
4 groups versus 5 groups	0.7025	0.3819	0.0250
7 groups versus 8 groups	0.9131	0.1091	0.0500

**Table 12:** Holm procedure on the effect of group size.

Dataset	*P*	*z*	Holm
4 members versus 10 members	0.0000	5.5646	0.0024
5 members versus 10 members	0.0000	4.6917	0.0025
4 members versus 9 members	0.0000	4.2552	0.0026
4 members versus 8 members	0.0000	4.1461	0.0028
6 members versus 10 members	0.0005	3.4915	0.0029
5 members versus 9 members	0.0007	3.3824	0.0031
5 members versus 8 members	0.0011	3.2733	0.0033
4 members versus 7 members	0.0032	2.9459	0.0036
7 members versus 10 members	0.0088	2.6186	0.0038
6 members versus 9 members	0.0291	2.1822	0.0042
4 members versus 6 members	0.0382	2.0731	0.0045
5 members versus 7 members	0.0382	2.0731	0.0050
6 members versus 8 members	0.0382	2.0731	0.0056
8 members versus 10 members	0.1561	1.4184	0.0063
9 members versus 10 members	0.1904	1.3093	0.0071
7 members versus 9 members	0.1904	1.3093	0.0083
5 members versus 6 members	0.2301	1.2002	0.0100
7 members versus 8 members	0.2301	1.2002	0.0125
4 members versus 5 members	0.3827	0.8729	0.0167
6 members versus 7 members	0.3827	0.8729	0.0250
8 members versus 9 members	0.9131	0.1091	0.0500

**Table 13:** Test parameters for experiment on the effect of Δ.

Parameter	Value
Number of runs for each experiment	500
Number of iterations	2000
Velocity clamping, *V*_max _	4
Range of inertia weight, *ω*	0.9–0.4
Learning factors	
*c*_1_	2
*c*_2_	2
Number of groups	5
Group's size	6

**Table 14:** Friedman test on the effect of Δ.

		1%	5%	10%	50%	100%
*f*_1_	Mean	0.3812	0.3944	0.3839	0.3932	0.3738
	Friedman rank	2	5	3	4	1

*f*_2_	Mean	0.0732∗*e* − 12	0.1061∗*e* − 12	0.0612∗*e* − 12	0.1925∗*e* − 12	0.1401∗*e* − 12
	Friedman rank	2	3	1	5	4

*f*_3_	Mean	38.4925	40.8531	39.8487	41.5781	40.5706
	Friedman rank	1	4	2	5	3

*f*_4_	Mean	0.3428∗*e* − 09	0.2885∗*e* − 09	0.9245∗*e* − 09	0.2542∗*e* − 09	0.2786∗*e* − 09
	Friedman rank	4	3	5	1	2

*f*_5_	Mean	0.2956∗*e* − 08	0.2979∗*e* − 08	0.3365∗*e* − 08	0.4385∗*e* − 08	0.4271∗*e* − 08
	Friedman rank	1	2	3	5	4

*f*_6_	Mean	0.1400∗*e* − 04	0.1180∗*e* − 04	0.1210∗*e* − 04	0.1339∗*e* − 04	0.1753∗*e* − 04
	Friedman rank	4	1	2	3	5

*f*_7_	Mean	0.0072	0.0069	0.0073	0.0070	0.0073
	Friedman rank	3	1	4.5	2	4.5

*f*_8_	Mean	44.8242	44.4277	44.0441	43.8860	44.2639
	Friedman rank	5	4	2	1	3

*f*_9_	Mean	0.3479	0.3515	0.3457	0.3505	0.3521
	Friedman rank	2	4	1	3	5

Average Friedman rank		2.67	3	2.61	3.22	3.5
